# Role of Phytoremediation in Reducing Cadmium Toxicity in Soil and Water

**DOI:** 10.1155/2018/4864365

**Published:** 2018-10-23

**Authors:** Pooja Mahajan, Jyotsna Kaushal

**Affiliations:** Department of Applied Sciences, Chitkara University, Rajpura 140401, India

## Abstract

Heavy metals are a noxious form of pollutants present in soil and water. A new plant-based solar energy driven technology, phytoremediation, emerges as eco-friendly and cost-effective approach to remove heavy metal from various media with the help of hyperaccumulating plant species. This review paper aims to provide information on phytoremediation and its mechanisms for heavy metal removal especially to focus on Cadmium (Cd) metal and highlights the role of various hyperaccumulating plants for Cd metal remediation in soil and water. It complies various field case studies which play the important role in understanding the Cd removal through various plants. Additionally, it pinpoints several sources and the effects of Cd and other technologies used for Cd remediation. This paper provides the recent development in mechanisms of Cd hyperaccumulation by different plants, in order to motivate further research in this field.

## 1. Introduction

In the present scenario, the most important concern of environmentalists is the alteration in biogeochemical cycles due to the variety of organic and inorganic pollutants released by manmade activities [1]. Along with the growth in industrialization, different remediation technologies were also coming into practice all-over the world to deal with different categories of pollutants. Among such pollutants, heavy metals are prime and critical contaminants in our surroundings. Heavy metals are continuing to exist for a prolonged period in nature as compared to other organic pollutants such as pesticides or petroleum by-products. So this makes the presence of heavy metals a matter of special concern. With the development of the global economy, different heavy metals in varying concentrations have gradually increased in environment thus resulting in degradation of the environment [2].

Heavy metals are highly noxious for all biotic components of the environment. Heavy metal contamination results either from the direct water source or through biomagnification. Sometimes in mining areas, high air concentrations also become a source of heavy metal contamination [3]. For instance, the Love Canal tragedy of the Niagara Falls in the USA explained the disastrous heavy metal effect on its human as well as animal population [4]. Several conventional technologies are being used for eradication of heavy metals, but these require a huge capital cost and have other disadvantages also. With the chemical method, not only heavy metals are eliminated but also valuable components of soil get degraded. Moreover, chemical methods generate a large amount of slurry and cost also per capita get increased [5].

A solution to this problem was suggested in terms of a new innovative eco-friendly technology known as phytoremediation which utilizes plants for treatment of pollutants. In literature, phytoremediation is mentioned as bioremediation, greener remediation or as botanical-remediation [6]. Another author defines phytoremediation as remediation of pollutants from the environment by converting those into less toxic form with the use of green plants [7]. According to Environment Protection Guide of USA, the term phytoremediation has been used since 1991 to publish different case studies where plants were utilized to remediate various types of contaminants [8]. Out of this broad category of pollutants, we have emphasized mainly on the remediation of Cd metal through phytoremediation technique due to its toxicity as detailed in this paper. We also compared other Cd remediation techniques with phytoremediation. This review paper also discusses the various mechanisms adopted by various plants to reduce Cd toxicity.

## 2. Phytoremediation

Plants practice different ways to remediate a wide range of contaminants in the environment. Certain plants act as “green livers” as they possess such worthy competence for the degradation of many adamant xenobiotics and act as the sink for noxious contaminants. This “solar driven” technology has the ability to remove contaminants such as heavy metals (As, Cd, Cu, Cr, Hg, Ni, Pb, Se, Zn, etc.), radioactive metals (Cs, Sr, U, etc.), and organic compounds (Benzopyrene pesticides (PAHs), Trichloroethylene (TCE), Trinitrotoluene (TNT), etc.). Phytoremediation plants must possess qualities like (1) rapid growth, (2) high biomass, (3) hairy and deep-root system, and (4) high bioaccumulation coefficient. Plants with extraordinary metal-accumulating power in their parts are described as hyperaccumulating plants. According to Baker, hyperaccumulating plants have the ability to uptake, translocate, assimilate, pile up, and tolerate high concentration of metals [9]. In literature, approximately 400 plant species have been reported as hyperaccumulators of different heavy metal.

Firstly, Brooks devised the term hyperaccumulator. According to him, hyperaccumulator defined as the plant which is accumulating efficiently Ni (approx. 1000 mg kg^−1^) in their upper portions [10]. If any plant was able to accumulate the heavy metal in its dry weight more than 0.1 % then it is termed as hyperaccumulator [11] and if 50% remediation ability in 24 hours then it is termed as a good phytoremediation agent [12]. But a hyperaccumulator should show tolerance to that heavy metal along with bioaccumulation of heavy metal. Hyperaccumulators should have the metal concentration of 0.001% (Hg), 0.01% (Cd and Se), 1% (Mn, Zn), and 0.1% (Al, Cr, Co, Cu, Pb, and Ni) of the shoot dry weight [13] (Backer and Brooks, 1989). A very few and most common hyperaccumulator are listed in Table 1.

**Figure 1:**
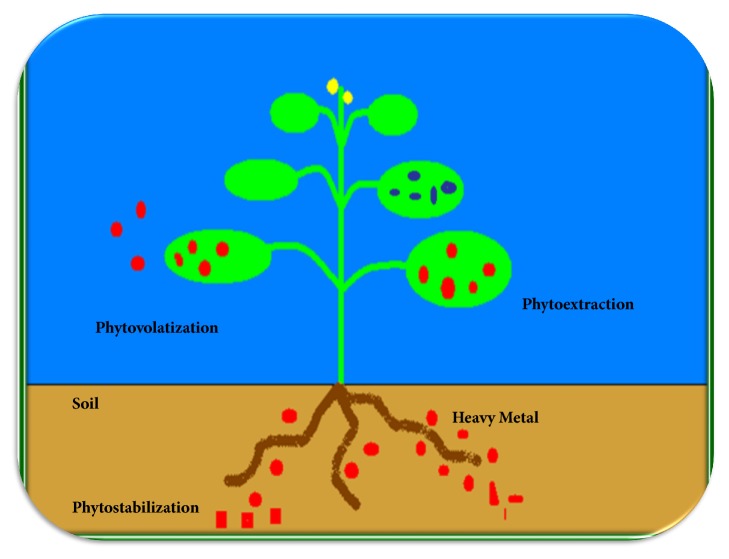
Phytoremediation mechanism adopted by plants to remediated heavy metals.

Hence, hyperaccumulator plants have mainly been reported from family Brassicaceae, Cunouniaceae, Caryophyllaceae, Asteraceae, Euphorbiaceae, Cyperaceae, Fabaceae, Lamiaceae, Violaceae, Poaceae, etc. [14]. Phytoremediation technology for heavy metal remediation involved different action mechanisms as shown in Figure 1.

### 2.1. Phytoextraction

Plants have the ability to phytoextract essential (Cu, Mg, Mo, K, Fe, Mn, Ni, P, and Zn) as well as nonessential metals (Se, B, Cd, Co, Cr, Ag, and Hg) required for plant growth. Nonessential metals are proven to be toxic to plants if present even in very low concentration and essential metals have also become noxious if present in more than the required quantity. In phytoextraction, plants ingest metals through roots and translocate the same to other parts. The main disadvantage of phytoextraction is that it is significant to only those sites which have low to medium amount of metal contamination as highly polluted sites prove to be noxious for the plant development [14]. The phytoextraction process depends mainly on the capability of the plant (1) to eradicate metal on fast pace (2) to accumulate maximum amount of metals in aerial parts (3) to tolerate high metal concentrations and (4) to grow fast [36, 37].* Pteris vittata* and* Chenopodium albums* have reported for phytoextraction of Arsenic and Lead, respectively [28, 38]. To increase bioavailability of metals, some chelating ligands like EDDS, EDTA, Succinic acid, Citric acid etc. were also added to contamination sites [18].

### 2.2. Rhizofiltration

Rhizofiltration mechanism is adopted by plants to remove heavy metals as well as radioactive metals like Cd, Cu, Ni, Pb, Cr, Cs, As, U, and Sr from aqueous solutions. In rhizofiltration, plant roots take up metal contamination from the wastewater streams or from wetlands. Suitability of the Plants for rhizofiltration depends upon the root system as roots filter metals from aqueous solution. Plants identified for adopting this mechanism have longer and hairy root systems of the considerable surface area. Indian mustard (*Brassica juncea*) and sunflower (*Helianthus annuus*) are favorable plants for rhizofiltration.* Brassica* effectively remediate Pb, Cd, Cu, Cr, Ni, Zn, and* Helianthus* rhizofiltered Ra and U [39].

### 2.3. Phytostabilization

Phytostabilization refers to the process in which a plant is able to immobilize metal in the resource and transform metallic toxic state to less toxic state. As a result migration of metals to other sites gets reduced [40]. Phytostabilization requires plants whose roots are able to develop into contamination zone and helps in immobilization of metal in soils either by root adsorption or by metal precipitation/ complexation/ reduction [41]. The highly noxious Cr (+6) gets transformed into Cr (+3), a less soluble and immobile form, through phytoremediation process [42]. Phytostabilization is found to be more effective in case of fine soils and high organic matter content [43]. Hence, phytostabilization does not even need removal of soil and disposal of contaminated biomass.

### 2.4. Phytovolatization

Phytovolatization is the eradication of pollutants by using plants converting the same to less toxic volatile form along with transpiration process using plants. Some organic pollutants and heavy metals such as arsenic, mercury, and selenium get volatilized by plants. In literature, macrophytes like* Chara canescens* (musk grass) and* Arabidopsis thaliana* were detailed for adopting phytovolatization [44]. Authors reported the eradication of Hg as Hg^2+^ ions which are less toxic forms of mercury. Tritium (the isotope of hydrogen) was stabilized as helium through phytovolatization [45]. Selenium found in the soil volatized as (CH_3_)_2_Se. This form of Se is 600 times less toxic than elemental Se [46].

## 3. Cd: Sources, Speciation, Toxicity, and Chemistry

Heavy metal Cd is widely distributed in water and soil as a nonessential toxic metal which occurs either in form of 0 or +2 oxidation state. It exists in nature as Cd (OH)_2_, CdCO_3_ and CdSO_4_. Cd also precipitates in the form of arsenates, phosphates, chromates, sulfides, etc. The permissible limit of Cd^2+^ in soil and plant is less than 1 mg L^−1^ and 0.005- 0.02 mg L^−1^_,_ respectively, according to USEPA [47]. The sources and permissible limit of Cd^2+^ in water are detailed in Table 2. The Cd concentration in water and soil resources gets increased day by day due to natural activities and anthropogenic activities [48].

**Figure 2:**
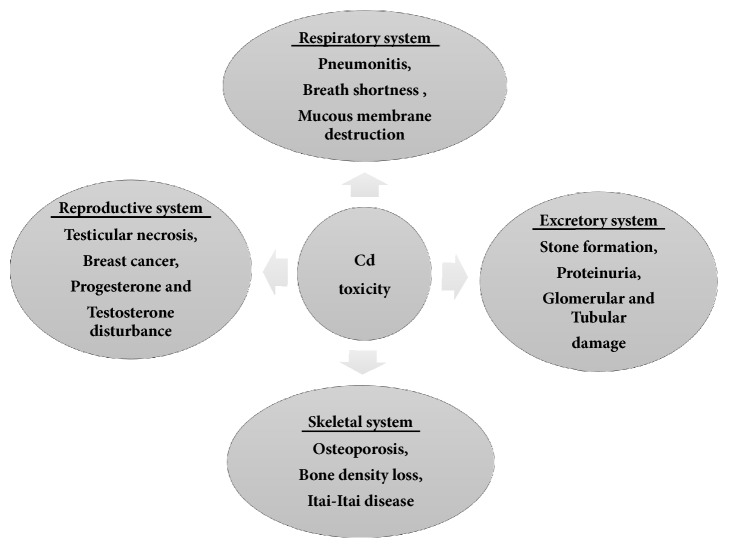
Cd effect on various organ system of human body.

Thus, the ecosystem gets contaminated either through direct Cd production or through secondary sources. It has been found that even a slight exposure to Cd results in the chronic effect on both animals and humans. In the human body, most of the Cd intake is through vegetable consumption [53]. An excessive amount of Cd dust causes multiple malfunctioning of organs (Figure 2).

Cd exposure to human bodies results in accumulation of Cd in the liver and kidneys which cause liver and renal malfunctioning and, on skeletal accumulation, results in Itai-Itai bone disease. A well-known case study on Jintsu river of Japan was due to Cd toxicity [54]. Once the Cd got accumulated in the human body, the estimation of its average half-life period is about 10 years [55] otherwise, in the environment, it is approximately 18 years [56]. Several physiological processes of plants like Nitrogen-metabolism and oxidative reactions were inhibited by Cd [57]. Presence of Cd in plants causes necrosis, leaf chlorosis, reduction in plant growth, and damage of photosynthetic machinery, especially photosystems PS-I and PS-II, which result into reduction in chlorophyll synthesis [58]. So, it is necessary to fetch an appropriate and a relevant solution to removal of Cd from the environment. Thus, the removal of nonessential metal such as Cd from environment becomes the area of interest for researchers.

## 4. Existing Techniques for Remediation of Cd

**Figure 3:**
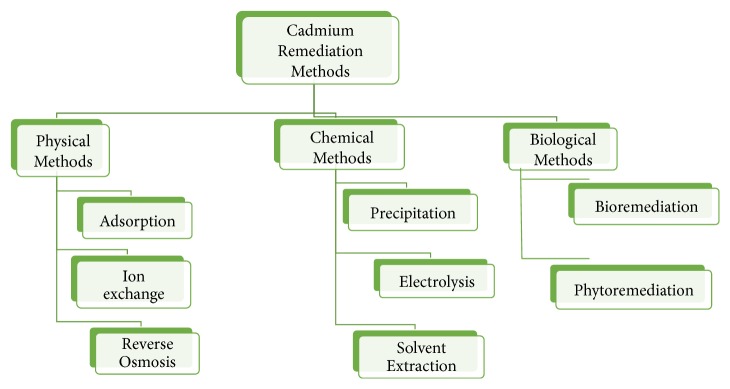
Flowchart of various methods used in Cd remediation.

The removal of Cd from contaminated soil and water can be achieved by various physical, chemical, and biological methods as shown in Figure 3. The wastewater treatment of industries and remediation of contaminated soil are still based upon the physical and chemical methods in spite of disposal problems and high cost.

### 4.1. Physical Methods

In physical methods, membrane filtration and adsorption are mostly used for toxic metal ions remediation process. Cd was mainly adsorbed via adsorbents such as activated Carbon, synthetic Al_2_O_3_, low-cost oxides/hydroxides of Al, Mg, or Fe, and waste product of agriculture [59–61]. High loading capacities adsorbents (> 90 mg g^−1^) such as silicate, wheat bran, fig leaves, pea peel, rice husk, sugarcane bagasse, baker's yeast, etc., also helped in remediation of Cd [62–65]. Particular membranes were also detailed to adsorb Cd from its aqueous solution such as simple liquid membranes [66], liquid membranes formed on support [67], emulsifying membranes, etc. [68]. An electrodialysis cell which was divided into five compartments has been also used for the removal of Cd from wastewater [69]. Ion exchange method was also devised by using Lewatit TP 260 cationic exchanger resin [70]. But in literature, there is lack of knowledge for safe disposal and reuse of loaded adsorbents. Hence, applications of adsorbents have still not been possible commercially.

### 4.2. Chemical Methods

In chemical methods, firstly Schlage Lock Company demonstrated a method in which addition of Barium acetate coagulated Cd from electroplating industry effluents [71]. In a precipitation process, Cd^2+^ ions get removed by addition of NaOH [72], Ca(OH)_2_ and Mg(OH)_2_ [73]. Some researchers also proposed cementation processes for Cd^2+^ ions removal from its aqueous solution [74]. Through solvent extraction technique, Cd^2+^ ions get extracted by using various extracts such as Cyanex 301, aqueous nitrogen donor ligand [75] and phosphorus based extract [76]. In a stripping step of solvent extraction, a large amount of solvent gets utilized during the process which is the major cause for the failure. So, the adaptation of such methods should not be advisable where heavy metal removal concentration was very less.

### 4.3. Biological Methods

The bioremediation of Cd through microorganisms such as bacteria [77], fungi Aspergillus [78], yeast species [79], green algae* Chlorella emersonii* [80], brown algae* Fucus vesiculosus* [81], etc. was well reported in the literature. Microbial remediation of Cd provides an effective way to render Cd toxicity but the growth of microbes is possible only in optimum climate conditions. This parameter restricts the use of microbes for remediation purpose. In last decade, another biological technique which has been proposed for Cd removal from contaminated soil and water resources is phytoremediation which is well suited, cost-efficient, and eco-friendly in comparison to the above-mentioned techniques of remediation. The present review is intended to give information with respect to phytoremediation of Cd.

## 5. Phytoremediation of Cd in Contaminated Soil

**Figure 4:**
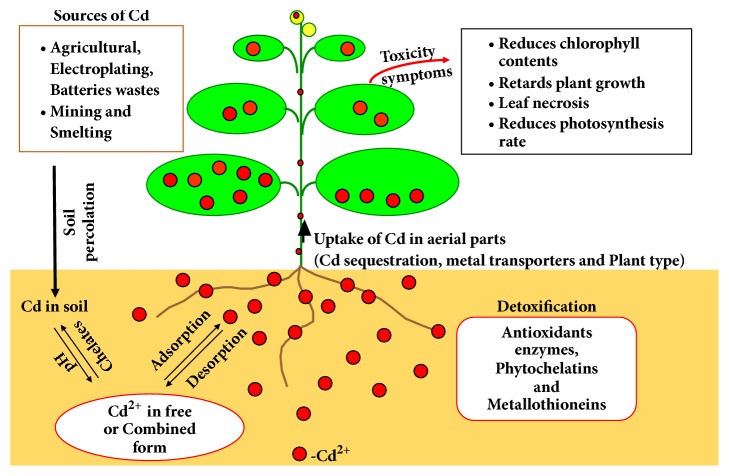
Phytoremediation mechanism of Cd adopted by soil plants.

Remediation of Cd-contaminated soil is a substantial problem around the globe and it became more significant due to the transfer of Cd in higher trophic levels of food-chain. Cd hyperaccumulators are of particular interest because of their ability to tolerate and take up significant amounts of heavy metal from soils. Plants of different species have different capabilities to hyperaccumulate Cd. As Cd has low affinities with soil ligands because of its mobile nature and hence, is easily extracted by roots and further transported to other aerial portions of the plant [82]. The factors responsible for remediation of Cd by plants are pH, temperature, its concentration in media, and even concentration of elements other than Cd [83]. The phytoremediation mechanism for Cd removal in soil plants is represented in Figure 4.

In literature, it was mentioned that plant species which are known as Cd hyperaccumulator have the ability to accumulate 10^5^ mg g^−1^ Cd in shoot dry weight [13]. A number of plant species have been reported for hyperaccumulation of Cd in soil as mentioned in Table 3.

*Thlaspi caerulescens* reported for Cd hyperaccumulation in the early 1990s.* T. caerulescens* showed much greater tolerance to Cd, with toxicity symptoms appearing at the 200 *µ*M concentration. The translocation of Cd from solution to upper portions and its concentration of shoots of* T. caerulescens *was remarkably high [84]. The hairy root culture of* T. caerulescens *also showed remediation of Cd from its aqueous solution [116]. These results confirmed* T. caerulescens *as a hyperaccumulating plant for the remediation of Cd pollution.* A. halleri* and* T. caerulescens *were found to hyperaccumulate Cd along with Zn [117]. In case of* T. caerulescens, *most of Cd accumulated in roots while in case of* A. halleri*, it was observed in leaf mesophyll [85]. But the problem found with these two plants* T. *caerulescens and* A. halleri *was that they were low-biomass plants and unable to bear an extensive range of environmental conditions. Consequently,* Calamagrostis epigejos, Sedum *species*, Brassica *species*, and Solanum nigrum* proposed as an alternative to* T. caerulescens *and* A. halleri* [118–125].

*C. epigejos* is a fast growing plant and able to tolerate extreme weather conditions and easily grown in poor sandy soils and marshy wetlands. Due to its high tolerance towards heavy metals, it was explored for Cd uptake and found low root to shoot transfer which infers that more ecological benefit of the plant in terms of phytostabilization can be achieved in comparison to phytoextraction [118].

In addition,* S. nigrum* have also been reported having the accumulation of high concentration of Cd along with Cu and Zn [96]. A study on EDTA effect on Cd uptake by* S. nigrum* was also reported. It was claimed that only moderate dose of EDTA 0.1 g Kg^−1^ in soil effectively enhanced phytoextraction of Cd whereas high dose 0.5 g Kg^−1^ adversely affected the growth of the plant and reduced biomass which results into reducing the effectiveness of phytoremediation method [119]. In a further study, the flowering stage potential of* S. nigrum* has been explored [120]. Thus, all these studies revealed that* S. nigrum *considerably accumulates a great amount of Cd and assists in controlling pollution in Cd-contaminated soils.

Another plant* Sedum alfredii *also showed a substantial potential for Cd remediation. In this study, it was shown that the amount of Cd gets enhanced on exposure to Zn concentrations [92]. The amount of both metals gets increased in leaves and stems with increase in concentration of Cd and Zn. This result established that* S. alfredii* works as hyperaccumulator of both the metals, Cd as well as Zn. The amendments such as humic acid and compost in soil with the DC current supply enhanced Cd extraction two- three folds by* S. alfredii* [121]. Another species* S. plumbizincicola *also reported to enhance Cd and Zn concentration on addition of EDTA by reducing mobility of ions in contaminated soil [122].

The large sized* Brassica juncea *(Indian mustard) was also found to phytoextract comparable amount of Cd as* T. caerulescens*.* B. juncea *plants have been found to tolerate inordinate Cd stress as compared to a Cd-sensitive species [123]. Another species of Brassica,* B. napus, *was found to be more stable on exposure of Cd as lipid changes were observed in cell membranes of* B. napus* on direct exposure to metal [124].* B. pekinensis* which is also called Chinese cabbage was also explored for Cd extraction from soil and its six different varieties were found to extract a significant amount of Cd [125].

Researches were also conducted in hydroponic systems to explore more efficient soil plants for Cd remediation. Experiments were conducted in soil as well as in hydroponic system to explore the phytoremediation potential of* Arundo donax*. The authors concluded that a significant and better uptake of Cd was observed in the hydroponic system as compared to soil cultures as Bio Concentration Factor (BCF) and Translocation Factor (TF) were more than 1 but on high exposure of Cd; antioxidant stress was shown by the plant [126]. Cd hyperaccumulation also reported in the bulb, shoot, and root of* A. sativum* (garlic) grown in hydroponic system and studies proved the capability of garlic to extract Cd from its solution and transport and store the same into various parts of garlic. With concentration increase of Cd^2+^, the amount of Cd in garlic roots gets enhanced. It has been found by investigators that the plant was able to extract Cd about 1826 times more than the control but a very limited quantity of Cd gets aggregated in bulbs and shoots of garlic [20]. Currently, Bidens pilosa was identified as Cd hyperaccumulator which accumulated 405.91 mg kg^−1^ and 1651.68 mg kg^−1^ in its shoots when grown in soil and nutrient solution, respectively. These results implied that concentration of Cd accumulated by Bidens pilosa grown in nutrient solution was much more than plants grown in soil. This study also revealed that the Cd translocation and accumulation in plant was controlled by K^+^ relative permeability ratio, MDA (Malondialdehyde) levels and conductivity of ions [100].

Recently,* Coronopus didymus, and* Abelmoschus manihot were among newly discovered plants for the hyperaccumulation of Cd in hydroponics. In* C. didymus, *TF reported to be higher than BCF [127]. In A. manihot, BCF values exceeded the reference value and TF values were also found to be greater than 1 on Cd treatment at 15–60 mg kg^−1^ [128]. It has been also reported in both the studies that superoxide anion amount, H_2_O_2_ content and antioxidative activities in roots and shoots get enhanced on exposure of a high dose of Cd which helps in the detoxification process [127, 128]. Hence,* C. didymus *and A. manihot can be used as Cd hyperaccumulator to remediate Cd from actual field sites.

## 6. Phytoremediation of Cd in Wastewater

The waste waters from industries are usually discharged into water bodies and aquatic macrophytes provide a way out for removal of heavy metals present in water.* Eichhornia crassipes, Alternanthera sessilis, Ceratophyllum demersum, Azolla pinnata, Chara coralline, Hygrorrhiza aristata, Hydrodictyon reticulatum*,* Hydrocotyle umbellate*,* Lemna minor, Salvinia, Pistia, Spirodela polyrhiza, Vallisneria spiralis,* etc. were some species of aquatic plants reported for heavy metal remediation from water bodies [129].

Phytoremediation experiments with* Eichhornia crassipes* which is commonly known as water hyacinth were well documented for the Cd removal along with Zn and Cr [130, 131]. Initially, Woverlton and McDonald reported the* E. crassipes potential *for heavy metal remediation in aquatic media. According to reports,* E. crassipes *was able to accumulate a substantial amount of Cd 371 and 6,103 mg kg^−1^ in shoots and roots (dry weight), respectively [32]. But in another study, it was observed that high concentration Cd (100 mg L^−1^) with other metals results in lesser amount of Cd in the aerial parts rather than in shoots [132]. Thus, from the perspective of phytoremediation,* E. crassipes *becomes a favorable choice among various macrophytes for remediation of wastewater effluent [133].

An interesting observation is reported by another author in case of* Hydrilla verticillata*. A submerged aquatic plant,* H. verticillata,* showed maximum absorption of Cd at the growth temperature (15-25°C), but in between 5 pm to 5 am it released some of its absorbed metal content in solution which, otherwise, showed a decline during the daytime [134].* Azolla pinnata, *another floating macrophyte, was found to be more effective in comparison to* E. crassipes. *The BCF for Cd in roots of* Azolla *was reported as 24,000 which was quite high [135]. At very low concentrations,* A. pinnata* and* L. minor *were found to be very effective in Cd remediation [19, 136, 137].* Pistia stratiotes *with long feathery roots was able to bear 20 mg L^−1^ Cd and plant growth got declined by increasing Cd concentration [138]. In comparison with* Salvinia herzegoii, *it accumulate a high level of Cd [139]. But another species of Salvinia,* S. minima* was reported as considered as a Cd hyperaccumulator. Hyperaccumulation of* S. minima *has been attributed to the increased specific surface area of roots with hydroxyl and carboxyl groups [140].

Another aquatic macrophytes such as* Potamogeton natans, Myriophyllum aquaticum*,* Wolffia globosa,* and* Typha *also showed the high accumulation of Cd [141–143]. Thus, the potential of aquatic macrophytes was studied very extensively for removal of Cd. The potential of these aquatic macrophytes can be used to remediate Cd from contaminated water streams in an eco-friendly manner.

## 7. Field Studies on Cd Remediation

All above-mentioned studies demonstrated the ability of hyperaccumulators of Cd in contaminated soil and water. In spite of this, a very few field trials were reported for phytoremediation of Cd metal. A case study was done at the El-Gabal El-Asfar region (GA region) of Cairo to investigate the role of the* S. nigrum *as metal hyperaccumulator in remediation of agricultural soils, which had been irrigated with sewage water and had got contaminated with heavy metals. A relative ratio of soluble sugars, alkaloid, phenolic compounds, proteins, and amino acids such as proline, glycine, etc. increased on enhancing the concentration of metal in the root, leaves, and stem of* S. nigrum *[144].

Another case study was carried out in agricultural fields of Mae Sot District, Thailand. These fields of Mae Sot were highly contaminated with Cd metal and it became a health issue of Thai people. Five different plant species* Chromolaena odorata, Gynura pseudochina, Conyza sumatrensis, Nicotiana tabacum *and* Crassocephalum crepidioides* developed and out of these except* Chromolaena odorata*, all other four species successful in removing Cd from the soil of agricultural fields [145]. Recently, it has been found that Napier grass reduced Cd concentration of soil by 4.6% in sites of Kyushu (Japan) where field trials were done in Cd- contaminated soil twice a year. There was no effect on yield of the crop but amazingly concentration of Cd from soil got increased at the second time cultivation [146]. In another study, three species of Armeria plant were explored for phytoremediation potential of the heavy metal in the minning area of Serbia. Three tested Armeria species were grown in eight different areas and none of the species showed shoot hyperaccumulation potential for any of the tested heavy metals. Armeria plant signified as root accumulators by authors due to their high bioconcentration factor 134 (Zn), 148(Cr), and 9 (Cd) in their roots [147].

Palutoglu et al. explored the phytoremediation potential of native species of Turkey in the Gümüsköy mining area which is known for the largest silver deposit. In this area, the concentration of Cd in contaminated soil was observed to be high 82.8 mg kg^−1^. The native plants under study showed 55.4 mg kg^−1^ Cd accumulation in their root and 43.5 mg kg^−1^ shoot, respectively. The plant species* Carduus nutans* and* Phlomis* were found to be the most effective out of eleven native tested species [148]. In a recent field study with Ganges ecotype of* T. caerulescen*, the role of soil geochemical factors and plant-soil interactions for Cd uptake were highlighted by hyperaccumulating plants [149]. This points towards the importance of the need for understanding site-specificity containing metal and soil geochemical properties in consideration before phytoremediation of actual field sites.

## 8. Uptake Mechanism and Detoxification of Cd in Plants

A comprehensive study of Cd detoxification and accumulation mechanism in plants was done by different researchers. Cd hyperaccumulating plants adopted various cellular and molecular mechanisms for their detoxification. Hyperaccumulation of Cd basically involves three processes, namely adsorption, transportation, and translocation. Adsorption of Cd primarily occurs through roots of the plant. Some factors like pH, humic acid, and medium are mainly responsible for effective absorption of Cd^2+^ [150]. In roots, the tissue in the root tip which adsorbed cations from the source. In the presence of root hairs, the efficiency of adsorption processes gets enhanced as the area of contact gets increased which accelerate the pace of Cd ion adsorption via root tissues [151]. In fact, root hairs were considered as the most influential part of root for adsorption process and the adsorption of most of the Cd from the soil takes place through cells of root hairs.

**Figure 5:**
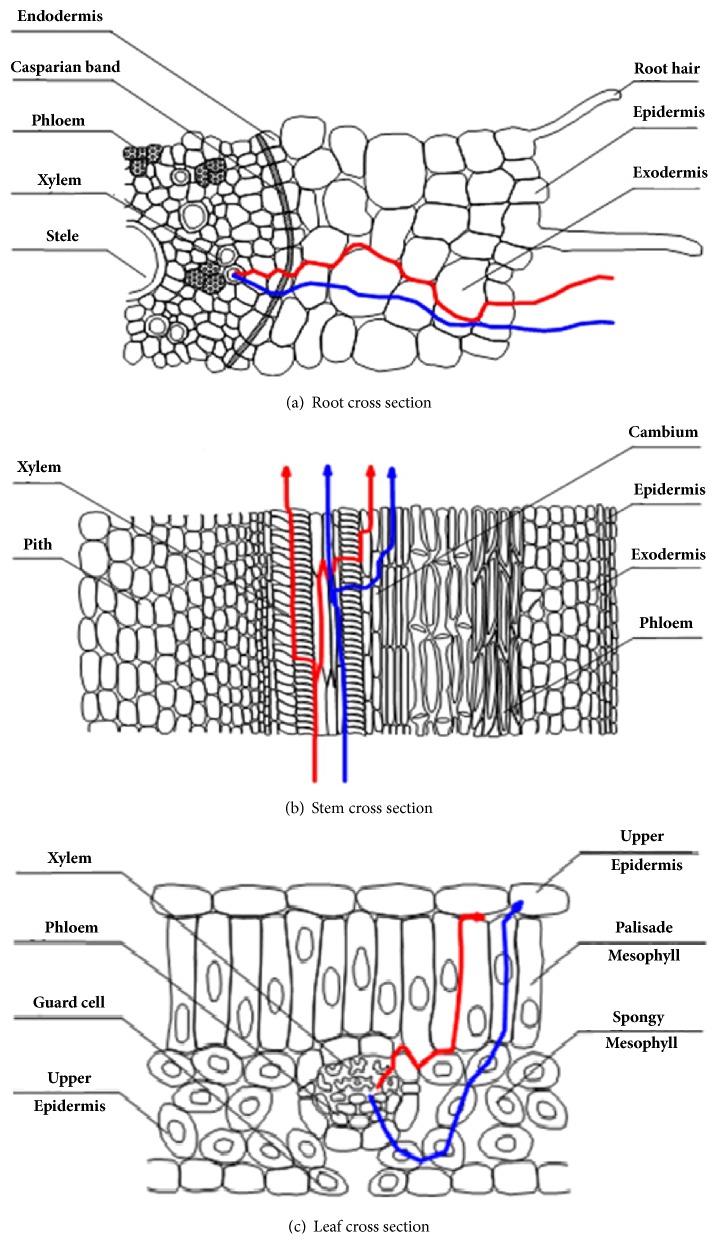
Diagram of apoplastic and symplastic pathways of Cd transport. The red and blue line show the apoplastic and symplastic pathway, respectively (source: Song et al. reprinted with permission).

Cd entry into the plant through root cells mainly takes place through the exchange of ions, the release of organic acids, chelating to metal ions and sequestration to root cells. The transportation of Cd depends on medium, metal and plant properties. Song et al. suggested that transportation of Cd can take place through apoplastic and symplastic pathways [152]. A diagrammatic representation of these pathways was shown in Figure 5. A prompt exchange of Cd^2+^ ions takes place with H^+^ in plasma membranes of root cells and through apoplast pathway, Cd^2+^ ions get adsorbed [153]. Another pathway for Cd entry in plant cells was through the symplast pathway. In this pathway, Cd gets combined with transporter proteins and then is passed via ion channels and enters into the epidermis layer of root cells [152]. But the relationship between the apoplastic and symplastic pathway of Cd transportation is not reported yet. In some cases, it has also been observed that plant roots also released chelates which bind with Cd^2+^ to form metal-ligand complexes about quick adsorption. The order for Cd accumulation in plants was found to be: roots > stems > leaves > fruits > seeds.

The mechanism of accumulation and antioxidative metabolism to tolerate Cd by hairy roots of* T. caerulescens* revealed that Cd metal-induced stress in tissues [116]. The factors like pH and H^+^-ATPase inhibitor enzyme affected Cd hyperaccumulation. The author also studied the Cd distribution in mesophyll protoplast of leaf cells in both the hyperaccumulating plants, namely,* T. caerulescens* and* A. halleri*. It was also suggested that a regulation mechanism existed on leaf mesophyll protoplasts in plasma membranes. Preexposure of Cd to the plant showed an exponential elevation in its concentration in leaf mesophyll protoplast of* T. caerulescens*, but a decline in the quantity of Cd in* A. halleri *[117]. From these results, it can be specified that the regulation mechanism for Cd transportation in plants is different for each plant. According to another study on* A. thaliana* for the remediation of Cd with the help of yeast protein, the yeast protein detached Cd through its extraction from source and transportation into vacuole of cells [154].

Scanning Electron Microscopy (SEM) and energy dispersive X-ray (EDX) microanalysis were also used by the researcher to analyse plant tissues of the lower epidermis, mesophyll protoplasts, and cell walls. SEM and EDX studies confirmed the Cd presence inside the epidermal cells as well as in the cell walls of plant tissue. Cd was found both in the large as well as small epidermal cells and not only in the cell walls but also in the cytoplasm of cells. These results concluded that metal is stored not only in one part of the cell but also gets distributed in other compartments of leaf mesophyll. It was also concluded by the author that in epidermal cells metabolic activities are almost negligible and Cd is mainly stored in these less active cells and hence does not interfere with activities of other cells [155].

Cd detoxification in hyperaccumulating plants occurred either through vacuole sequestration or by binding through cysteine-rich proteins. Plant sequestration of Cd^2+^ into the vacuole and removal of Cd^2+^ from the cytosol of the cell were also reported [156]. As vacuole of the cell is considered for detoxification process and a large number of metabolites get stored in it to detoxify the cytosol [157]. Vacuole sequestration of Cd has been reported to occur mainly through transporters Ca^2+^ exchangers (CAXs) and heavy metal ATP ase (HMAs) [158]. In Arabidopsis plant, HMAs is responsible for sequestering Cd in the root and controls Cd transfer from root to the aerial parts of plant [159]. Plants such as* S. alfredii *and* N. caerulescens* have potential to store a large amount of Cd in the aerial parts which reported to possess some high expression genes which play an important part in the accumulation of Cd [160]. The HMAs from both plants possessed high substrate specificity for Cd over other heavy metals like Zn, Pb, and Co [160, 161]. However, the substrate specificity of HMAs mechanisms of Cd hyperaccumulation has yet to be explored.

**Figure 6:**
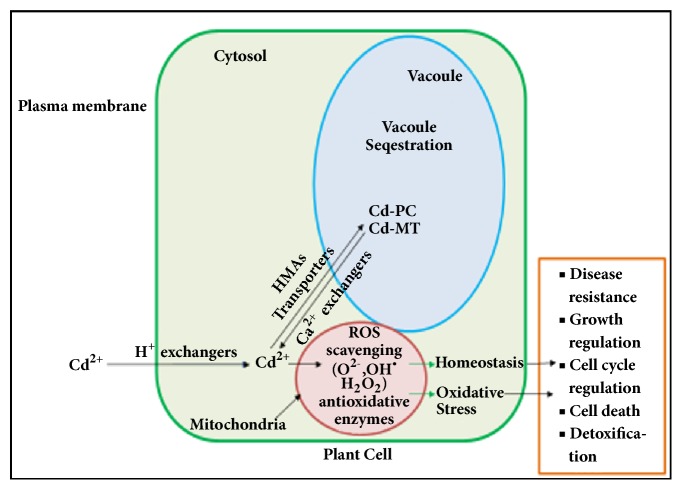
Schematic representation of Cd detoxification in plant cell (heavy metal ATP ases (HMAs), phytochelatins (PC), metallothioneins (MT), and reactive oxygen species (ROS)).

Another detoxification Cd mechanism involved two types of cysteine-rich peptides known as Phytochelatins (PCs) and Metallothioneins (MTs) [162]. Being a thiol reactive metal, Cd bound with these peptides gets detoxified. MTs are low molecular mass peptides which amalgamated on ribosomes. Plants with complex MTs genes are able to tolerate the toxicity of metal ions and aid transportation the ions as well. On exposure to Cd, MTs are also helpful in the shielding of chloroplasts of guard cell from degradation [163]. When detoxification of Cd occurs through PCs then MT bind Cd as such in the cytoplasm and the same was not tucked away into the vacuole. PCs were also found as Cd-binding peptides through carboxyl and the sulfhydryl residues in presence of enzyme phytochelatin synthase (PCS) and Cd thought to be acting as cofactor for glutathione to PCs conversions [163]. Also, various types of reactive oxygen species (ROS) such as superoxide anion O^2-^ and H_2_O_2_ and antioxidative enzymes induced detoxification process of Cd at high Cd concentrations [127, 128]. A schematic representation of Cd detoxification in plant cell is given in Figure 6. Recently, genome-wide studies have been also used to explore detoxification mechanisms in Cd metal hyperaccumulators* T. caerulescens *and* Brassica chinensis* [161, 164] but still, there is a lot of scope of research.

## 9. Conclusion

Cd removal through phytoremediation emerges as a sustainable technology for contaminated soil as well as wastewater. Phytoremediation has high performance results when compared with other conventional technologies for Cd metal removal. The plant species from wide group of families have been recognized as Cd hyperaccumulators in last two decades. Different hyperaccumulating plants have varied abilities to accumulate, sequester, and detoxify Cd. Research studies are in headway to elucidate the various mechanism adopted by different plants to combat the toxicity of Cd at physiological and molecular level. But, the genetic level control of Cd detoxification in plants is not yet identified. Despite the lot of developments made in field of Cd phytoremediation from contaminated soil and water, only a limited number of research studies have taken place in field conditions. Hence, there is an urgent need for research on improving experimental design of phytoremediation relevant to Cd concentration in soil and water. In addition, the methods for the disposal of Cd-enriched biomass need to be further explored. Moreover, for practical approach, there is urgent need of integration of established method with phytoremediation technique to provide an innovative solution for Cd removal from soil and water.

## Figures and Tables

**Table 1:** Hyperaccumulators reported for phytoremediation of heavy metals.

Heavy metal	Plant	Mechanism	Medium	References
As	*Pteris vittata*	Phytoextraction	Soil	[15]
	*Piricum sativum*	Phytostabilization	Soil	[16]

Cd	*Oryza sativa*	Phytoextraction	Soil	[17]
	*Vetiver grass*	Phytostabilization	Soil	[18]
	*Lemna minor*	Rhizofiltration	Water	[19]
	*Allium sativum*	Phytoextraction	Hydroponic solution	[20]
	*Lemna minor*	Rhizofiltration	Water	[19]

Cr	*Brassica juncea*	Phytoextraction	Soil	[21]
		Rhizofiltration	Water	[22]

Hg	*Marrubium vulgare*	Phytoextraction	Soil	[23]
	*Pistia stratiotes*	Rhizofiltration	Water	[24]

Ni	*Alyssum lesbiacum*	Phytoextraction	Soil	[25]
	*Agropyron elongatum*	Phytostabilization	Soil	[26]
		Rhizofiltration	Water	[27]

Pb	*Chenopodium album*	Phytoextraction	Soil	[28]
	*Vetiveria zizanioides*	Phytostabilization	Soil	[29]
	*Hemidesmus indicus*	Rhizofiltration	Water	[30]

Se	*Brassica rapa L.*	Phytoextraction	Soil	[31]
	*Lemna minor*	Rhizofiltration	Water	[32]
	*Brassica spp.*	Phytovolatization	Water	[33]

U	*Lolium perenne*	Phytoextraction	Soil	[34]

Zn	*Cynodon dactylon*	Phytoextraction	Soil	[28]
	*Brassica juncea*	Rhizofiltration	Water	[35]

**Table 2:** Sources and permissible limits of Cd [49–52].

Natural Sources	Industrial Sources	Uses	Permissible limit (mg L^−1^)
Coal combustion, iron and steel production, phosphate fertilizer manufacture and use, and zinc production, volcanic activities	Zinc smelting, mining, waste batteries, e-waste, fuel combustion, manufacturing of alloys, pigments and dyes, textile operations etc.	Electroplating of steel, Ni-Cd batteries, cellular telephones, Laptop computers and camcorders	0.003 (IS 10500) 0.003 (WHO) 0.005 (USEPA) 0.005 (EU Standard) 0.002 (NHMRC, Australia)

**Table 3:** Cd hyperaccumulators reported for phytoremediation in soil.

Plant species	Cd Concentration (mg kg^−1^)	Hyperaccumulating portion	Reference
*Thlaspi caerulescens *	1140	Shoots	[84]

*Arabidopsis halleri*	281	Leaves	[85]
	1000	Shoots	[86]

*Brassica napus *	11.94, 263	Stems, Leaves	[87]

*Arabis gemmifera *	5600, 6643	Leaves, Shoots	[88]

*Arabis paniculata *	1662	Leaves	[89]

*Viola boashanensis *	1168	Shoots	[90]

*Salsola kali *	2075	Stems	[91]

*Vetiver zizanioides*	0.33	Leaves	[18]

*Sedum alfredii*	9000	Leaves	[92]

*Rorippa globosa *	150	Leaves	[93]

*Chromolaena odorata *	102	Shoots	[94]

*Iris lactea *	529	Shoots	[95]

*Solanum nigrum *	125	Leaves	[96]

*Phytolacca americana *	10,700	Leaves	[97]
	2840	Stems	[98]

*Bidens pilosa*	108-376, 144-400, 27.9-101	Stem, Leaves, Seeds	[99]
	405.91	Shoots	[100]

*Atriplex halimus*	218	Shoots	[101]

*Amaranthus mangostanus*	260	Shoots	[102]

*Amaranthus hybridus *	242	Shoots	[103]

*Picris divaricata *	1109	Shoots	[104]

*Gynura pseudochina *	457	Shoots	[105]

*Lonicera japonica *	345 and 286	Stems and Shoots	[106]

*Lycopersicon esculentum *	130	Shoots	[107]

*Arthrocnemum macrostachyum *	70	Shoots	[108]

*Prosopis laevigata *	8176	Shoots	[109]

*Carthamus tinctorius *	277	Leaves	[110]

*Helianthus tuberosus *	>100	Stems and Leaves	[111]

*Siegesbeckia orientalis *	193	Shoots	[112]

*Youngia erythrocarpa*	100	Shoots	[113]

*Macleaya cordata*	393	Plant	[114]

*turnip landraces*	52.94 -146.95	Shoots	[115]
